# Supplementary material for: Management of Stress Urinary Incontinence by Obstetricians and Gynecologists in Jordan: A Nationwide Survey Study
Source: Healthcare (Basel). 2024 Jul 27;12(15):1489. doi: 10.3390/healthcare12151489 (PMC11311483; doi:10.3390/healthcare12151489)
Supplement: Supplementary file 1 [file healthcare-12-01489-s001.zip › healthcare-3043737-supplementary.pdf]

# A Questionnaire to Study the Management of Stress Incontinence Among Gynecologists in Jordan.

**Our respected consultant**
**Good Day**
**We are a research group of medical students from the university Of Jordan- department of OBS/GYNE.**
**We are conducting this online questionnaire as a survey tool for studying the management of stress incontinence among Gynecologist/ Urologist in Jordan.**
**We trust your heartful cooperation and willingness to give genuine representative responses to all questions in the survey.**

**The data you provide will only be used for research purposes and will be dealt with confidentially. None of the questions address your identity, which will be anonymous throughout.**

**Proceeding in completing this questionnaire will be taken as consent to participate in this study, and you are always free to withdraw at any point.**

**A big thank you from all research team**

members.

* تشير إلى أنّ السؤال مطلوب

## Personal Information

1. Age*

*حدد دائرة واحدة فقط.*

< 30 years old

30–40 years old

41–55 years old

>55 years old

2. Gender*

*حدد دائرة واحدة فقط.*

Female

Male

3. Speciality*

*حدد دائرة واحدة فقط.*

Obstetrics/gynecology
Urogynecology
Urology
Other

4. Main practice setting*

*حدد دائرة واحدة فقط.*

Private sector
Prime Ministry of health
Academic
Other

5. Location of practice*

*حدد كل الإجابات الملائمة.*

- [ ] عمان
- [ ] الزرقاء
- [ ] إربد
- [ ] البلقاء
- [ ] عجلون
- [ ] جرش
- [ ] المفرق
- [ ] مأدبا
- [ ] معان
- [ ] الكرك
- [ ] الطفيلة
- [ ] العقبة

6. Fellowship/subspecialty training in urogynecology *

*حدد دائرة واحدة فقط.*

- Yes—completed
- Yes—not completed
- No

7. Years since finishing training*

حدد دائرة واحدة فقط.

- < 5 years
- Between 5 and 10 years
- > 10 years
- no fellowship/subspecialty training

## Management of urinary stress incontinence

8. 2. What is your preferred first line of management for urinary stress incontinence? *

حدد دائرة واحدة فقط.

- Pelvic floor muscle physiotherapy
- lifestyle and behavioural therapy
- Medications
- surgical procedures
- أخرى: ____________

9. 3. Before surgical correction for stress urinary incontinence, what investigation do you usually perform? *

*حدد دائرة واحدة فقط.*

- Multichannel urodynamics
- Stress test only
- Stress test and post-void residual
- Stress test, uroflow, and post-void residual
- Physical examination only
- Video urodynamics

10. 4. Would you repeat urodynamics before a second operations? *

*حدد دائرة واحدة فقط.*

- All of the time
- Some of the time
- None of the time

11. 5. What is your primary choice of surgery for stress incontinence? *

*حدد دائرة واحدة فقط.*

- Burch procedure
- Mid-urethral sling—retropubic kit
- Mid-urethral sling—transobturator kit
- Suburethral sling (biological)—allograft, xenograft, or autograft
- Single-incision slings (SIS)
- Bladder neck needle suspension
- Kelly plication
- Urethral bulking agent (UBA) injection

12. 6. Preferred surgical treatment for stress urinary incontinence with normal urethral pressure studies/normal urethral function and urethral hypermobility *

حدد كل الإجابات الملائمة.

- [ ] Burch procedure
- [ ] Mid-urethral sling—retropubic kit
- [ ] Mid-urethral sling—transobturator kit
- [ ] Suburethral sling (biological)—allograft, xenograft, or autograft
- [ ] Single-incision slings (SIS)
- [ ] Bladder neck needle suspension
- [ ] Kelly plication
- [ ] Urethral bulking agent (UBA) injection
- [ ] I do not use urethral pressure studies and/or urethral mobility

13. If you don’t use urethral pressure studies and/or urethral mobility. Skip questions 7& 8

7. Preferred surgical treatment for stress urinary incontinence with intrinsic sphincter deficiency/decreased urethral pressure studies and urethral hypermobility

حدد كل الإجابات الملائمة.

- [ ] Burch procedure
- [ ] Mid-urethral sling—retropubic kit
- [ ] Mid-urethral sling—transobturator kit
- [ ] Suburethral sling (biological)—allograft, xenograft, or autograft
- [ ] Single-incision slings (SIS)
- [ ] Bladder neck needle suspension
- [ ] Kelly plication
- [ ] Urethral bulking agent (UBA) injection

14. 8. Preferred surgical treatment for stress urinary incontinence with decreased urethral pressure studies/intrinsic sphincter deficiency and non-mobile urethra

حدد كل الإجابات الملائمة.

- [ ] Burch procedure
- [ ] Mid-urethral sling—retropubic kit
- [ ] Mid-urethral sling—transobturator kit
- [ ] Suburethral sling (biological)—allograft, xenograft, or autograft
- [ ] Single-incision slings (SIS)
- [ ] Bladder neck needle suspension
- [ ] Kelly plication
- [ ] Urethral bulking agent (UBA) injection

15. 9. Preferred secondary surgical treatment of stress urinary incontinence after failure of initial procedure (may choose more than one answer) *

حدد كل الإجابات الملائمة.

- [ ] Burch procedure
- [ ] Mid-urethral sling—retropubic kit
- [ ] Mid-urethral sling—transobturator kit
- [ ] Suburethral sling (biological)—allograft, xenograft, or autograft
- [ ] Single-incision slings (SIS)
- [ ] Bladder neck needle suspension
- [ ] Kelly plication
- [ ] Urethral bulking agent (UBA) injection
- [ ] Overlap of sling
- [ ] Tape tightening
- [ ] Refer to Urogynecologist
- [ ] Refer to Urologist

16. 10. Preferred surgical treatment for proven stress urinary incontinence when performing a surgery for any pelvic organ prolapse *

حدد دائرة واحدة فقط.

- Burch procedure
- Mid-urethral sling—retropubic kit
- Mid-urethral sling—transobturator kit
- Suburethral sling (biological)—allograft, xenograft, or autograft
- Single-incision slings (SIS)
- Bladder neck needle suspension
- Kelly plication
- Urethral bulking agent (UBA) injection
- Perform staged procedure at two separate times: repair POP followed by SUI surgery at a later date if indicated

17. 11. Preferred surgical treatment to prevent de novo stress urinary incontinence when performing a concomitant surgery for POP (no complaints of SUI and no USI on evaluation) *

حدد دائرة واحدة فقط.

- Burch procedure
- Mid-urethral sling—retropubic kit
- Mid-urethral sling—transobturator kit
- Suburethral sling (biological)—allograft, xenograft, or autograft
- Single-incision slings (SIS)
- Bladder neck needle suspension
- Kelly plication
- Urethral bulking agent (UBA) injection
- I do not perform any anti-incontinence procedure in this type of patient

18. 12. Material used for mid/sub-urethral sling *

*حدد دائرة واحدة فقط.*

- Autologous fascia
- Cadaveric fascia lata
- Synthetic material
- Biological graft

19. 13. type used for bulking agent (UBA)*

*حدد دائرة واحدة فقط.*

- Calcium hydroxyapatite (Coapatite)
- Polydimethylsiloxane (Macroplastique)
- Bovine collagen (Contigen)
- Carbon beads (Durasphere)
- Polytetrafluoroethylene (Teflon, PTFE)
- Fat
- Polyacrylamide (Bulkamid)

لم يتم إنشاء هذا المحتوى ولا اعتماده من قِبل Google.

نماذج Google
